# Prevalence of mandibular biradicular canines using cone-beam computed tomography. A cross-sectional study

**DOI:** 10.4317/jced.64125

**Published:** 2026-06-29

**Authors:** Carla Melani Mayan-Revollo, Jhoana Mercedes Llaguno-Rubio, Gustavo Adolf Fiori-Chíncharo, Luis Ernesto Arriola-Guillén

**Affiliations:** 1 Division of Oral and Maxillofacial Radiology, Faculty of Dentistry, Centro Universitário do Norte de São Paulo (UNORTE), São Paulo, Brazil; 2 Division of Oral Radiology, Instituto Latinoamericano de Altos Estudios en Estomatología (ILAE), Lima, Peru; 3 Division of Oral Radiology, Instituto Latinoamericano de Altos Estudios en Estomatología (ILAE), Lima, Peru; 4 Division of Orthodontics, Faculty of Dentistry, Universidad Científica del Sur, Lima, Peru

## Abstract

**Background:**

Mandibular biradicular canines correspond to a rare anatomical variant. The objective of this study was to determine the prevalence of mandibular biradicular canines using cone-beam computed tomography (CBCT) in order to enable timely recognition of this clinically relevant condition, as it influences the planning and success of procedures in specialties such as endodontics, orthodontics, oral surgery, among others.

**Materials and Methods:**

A cross-sectional and retrospective study was conducted analyzing 1130 CBCT volumes acquired with a Planmeca ProMax 3D MID scanner at a resolution of 200 µm, performed between October 2024 and October 2025 in a private imaging center in La Paz, Bolivia. The root morphology of mandibular canines was evaluated in patients older than 13 years in whom this area could be properly assessed. Three-dimensional evaluation was performed using Romexis 6 software in the multiplanar reconstruction window. Each canine was centered along its longitudinal axis, and a sweep was performed from crown to apex, in the coronal plane from buccal to lingual, and in the sagittal plane from mesial to distal, allowing visualization of root bifurcations. The presence of anatomical variants was then recorded. Fisher's exact test and Student's t-test were used, with a significance level of P &lt; 0.05.

**Results:**

The study revealed a prevalence of 1.7% for this condition. Women showed a higher incidence (2.3%) compared to men (0.8%). Most cases were unilateral, with a predominance of root bifurcation at the middle third. Notably, 100% of identified cases presented a buccolingual orientation of the roots, a finding of clinical importance due to the potential for structural superimposition.

**Conclusions:**

The results underscore the importance of considering this anatomical variant during the planning of dental treatments, particularly in endodontic, orthodontic, and surgical procedures, to avoid complications. Although its prevalence is low, recognizing this variant can contribute to improved treatment prognosis in affected patients.

## Introduction

A detailed understanding of root anatomy is an essential aspect of dental practice, as it directly influences treatment planning and the prognosis of multiple clinical procedures. Permanent mandibular canines typically present a single root and root canal; however, several uncommon morphological variations have been described, including the presence of two roots. This condition, known as a biradicular canine, has been reported in approximately 2% of cases worldwide, although prevalence varies among populations (1 - 3), with Spain showing the highest recorded rate (6.7%). Additionally, some reports indicate that the presence of two roots is more frequent in women (2 - 9). From an embryological perspective, root development begins once the crown has formed and the enamel reaches the future cemento-enamel junction. At this stage, the apical region of the enamel organ elongates, forming Hertwig's epithelial root sheath (HERS). This bilayered epithelial structure is positioned between the dental papilla and the dental follicle. HERS extends apically and plays a crucial role in guiding root formation, determining the shape, size, and number of roots. Any changes in HERS, whether due to overactivity or pathological degeneration, can lead to invaginations of the dental papilla. Consequently, this may result in the formation of additional roots or alterations in the morphology and structure of existing roots (4 , 5). The diagnosis of these anatomical variants is often incidental during routine radiographic examinations (6 - 12). However, root anatomy may present complex configurations, such as those reported by Okumus et al. (13) in a Turkish subpopulation, that may go unnoticed in 2D imaging. For this reason, cone-beam computed tomography (CBCT) constitutes an invaluable tool for their detection, as it enables three-dimensional assessment of root morphology without structural superimposition (6 - 9 , 13). Considering that the mandibular canine is a tooth of fundamental importance in the dental arch due to its strategic support in guiding masticatory function, as well as its role in providing occlusal guidance during eccentric movements, morphological variations such as the presence of two roots must be considered. This is essential for clinicians to consider when planning endodontic, orthodontic, or oral surgery treatments in order to avoid iatrogenic complications and preserve the functional integrity of the tooth (10 - 12). In this context, updating the prevalence of this condition is clinically relevant to support appropriate clinical decision-making. Therefore, the purpose of the present study was to determine the prevalence of mandibular biradicular canines, to improve knowledge of possible anatomical variant information that is crucial for successful professional practice to minimize the possibility of treatment-related complications-and to contribute valuable data to the literature, which remains scarce on this topic.

## Materials and Methods

**Figure 1:**
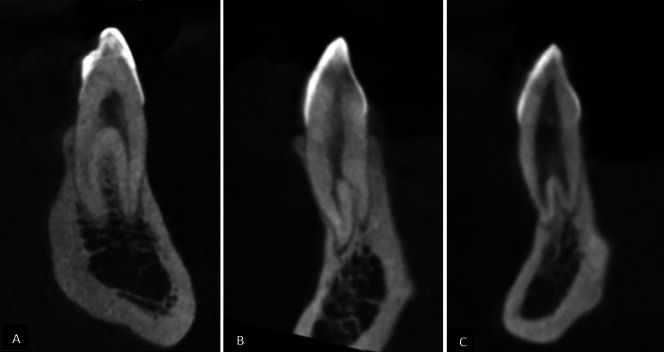
A) Sagittal section of tooth 3.3 shows a two-rooted canine at the cervical third of the root. B) Sagittal section of tooth 4.3 shows a two-rooted canine at the middle third of the root. C) Sagittal section of tooth 4.3 shows a two-rooted canine at the apical third of the root.

- Study Design and Ethical Approval This experimental study received ethical approval under registration number 007/2026 from the Division of Oral and Maxillofacial Radiology, Faculty of Dentistry, Centro Universitário do Norte de São Paulo (UNORTE), São Paulo, Brazil. - Population and Sample This cross-sectional, observational, and retrospective study was approved by the Northern University Center of São Paulo, Brazil. The sample consisted of 1,130 CBCT volumes from individuals over 13 years of age, with recorded age and sex, obtained from a private imaging center in the city of La Paz, Bolivia, between October 2024 and October 2025. Sample size calculation was performed using Fisterra software (https://www.fisterra.com/), with a 95% confidence level, 1% precision, an expected proportion of 2.83% (based on a previous pilot study), and a 15% margin of error, resulting in a minimum required sample size of 1,118 volumetric tomographic scans. Selection criteria included CBCT volumes that allowed adequate evaluation of the study area and that presented bilateral mandibular canines. Exclusion criteria included CBCT volumes with artifacts, ghost images, or other imaging distortions that prevented accurate assessment of the region of interest, as well as scans from patients with pathologies and/or a history of craniofacial syndromes that could alter root morphology. - Image Acquisition All scans were obtained using a Planmeca ProMax 3D MID CBCT unit, at a resolution of 200 µm, 90 kV, 8 mA, and 12 s, following the manufacturer's recommended protocol. During image acquisition, each patient was firmly positioned in the head support, maintaining the Frankfort plane parallel to the floor, with the canine guide aligned to the mid-level of the maxillary canine's longitudinal axis, and a bite separator placed between the maxillary and mandibular incisors, (Fig. 1).

1

- Image Evaluation Three-dimensional evaluation was performed using Planmeca Romexis 6.0 specialized software and viewed on a 24-inch AOC LCD monitor (model I2769Vm) connected to an ASUS desktop computer equipped with an Asus Strix Z270 Gaming motherboard, an Intel Core i7-7700 processor (6.6 GHz), and an EVGA GTX 1060 graphics card. Image interpretation was conducted in a controlled environment measuring 4 × 3 m, with dim natural lighting (25 KW), during morning hours from 9:00 to 11:00 a.m. The evaluator was positioned 40 cm from the monitor, seated in an ergonomic chair that maintained spinal alignment between 60° and 90°. In the multiplanar reconstruction window, each mandibular canine was centered along its longitudinal axis. A systematic sweep was then performed from crown to apex, with coronal plane evaluation from buccal to lingual and sagittal plane evaluation from mesial to distal, enabling visualization of root bifurcations. The presence of anatomical variants was subsequently recorded. All information was documented in a data collection sheet (Microsoft Excel template) according to the operationalization of variables. Anonymity of all individuals was ensured in accordance with the guidelines of the Declaration of Helsinki. - Statistical Analysis SPSS 19 for Windows was used for data analysis. The statistical analysis included Student's t-test and Fisher's exact test. A significance level of P &lt; 0.05 was applied.

## Results

The initial characteristics of the sample are summarized in Table 1.

Table 1

The analysis of biradicular canines is presented in Table 2, which indicates that this condition occurred in 1.7% of cases.

Table 2

Women had a higher prevalence of 2.3% compared with men (0.8%; p = 0.045). However, no significant association was found between the presence of a biradicular canine and the affected side (p = 0.305). Most of the affected cases were unilateral, and there was no significant association with sex (p = 0.554). In terms of furcation levels, a majority of cases were located in the middle third in women (85.7%), while 75% of men also exhibited this level, with 25% apically positioned (p = 0.044). Notably, all cases (100%) displayed a buccal-lingual position of the biradicular canine, regardless of sex (Table 3).

Table 3

## Discussion

The occurrence of mandibular biradicular canines continues to be an uncommon, though not negligible, anatomical finding. Therefore, a comprehensive understanding of root anatomy and its morphological variations is essential for achieving successful clinical outcomes. Endodontists, orthodontists, and oral surgeons frequently perform procedures involving these teeth, making knowledge of the frequency of this variant clinically relevant. For this reason, the present investigation sought to determine how many individuals in the studied population present this condition. In this study, the prevalence of mandibular biradicular canines was found to be 1.7%, a value consistent with the range reported in the international literature, where average prevalence is estimated between 2% and 5% (1 , 3). Variations according to geographic region have been described. Lambrianidis et al. (14) suggested that these discrepancies may be related to differences in sample size and racial characteristics. For example, studies have reported a prevalence of 0% in South India, 0.7% in China, 1.2% in Malaysia, 1.3% in the Iranian population, and 1.7% in Brazil, similar to the prevalence found in the Bolivian population assessed in this study (15 - 19). Spain has shown the highest prevalence reported to date, at 6.7% (6 , 7). In our study, a higher prevalence was observed in women (2.3%) compared with men (0.8%) (p = 0.045). This finding is consistent with multiple international studies that also reported a female predominance in the occurrence of mandibular biradicular canines (16). Several authors have suggested that this pattern may be explained by genetic variations and differences in dental developmental processes that could influence morphogenesis (2 , 7 , 9). This phenomenon underscores the importance of considering sex as a relevant factor in dental anatomy research (20) and in the planning of dental procedures. Although no specific studies have yet described the mechanisms underlying sex predilection in this variant, it continues to be regarded as a multifactorial tendency, not attributable to a single cause but rather to a complex interaction of factors involved in tooth development (21). Regarding laterality, the results of the present study are consistent with previous investigations showing that anatomical position, whether on the right or left side is not a determining factor in the manifestation of this morphological variant. No significant lateral preference was observed; however, given that most cases were unilateral, these findings align with reports in the literature indicating a low frequency of bilateral cases (1 , 3). Likewise, the reduced incidence and the limited number of reported bilateral presentations suggest that, although the variant may occur on both sides of the dental arch, its nature tends to be isolated and asymmetrical and is not associated with a duplication mechanism (16 , 22). A clinically relevant finding of this study was that 100% of the cases exhibited a buccolingual orientation of the roots. This is particularly important in clinical practice because this pattern can go unnoticed in two-dimensional radiographs due to structural superimposition. This observation highlights the importance of cone-beam computed tomography (CBCT), which enables three-dimensional visualization and analysis of this anatomically complex and difficult-to-detect variant (13 , 23 - 25). In mandibular canines with two roots, bifurcation may occur at different root levels, which implies varying degrees of clinical difficulty depending on its location. This study identified a marked predominance of bifurcation in the middle third of the root, with a frequency of 85.7% in women and 75% in men. This distribution suggests that root morphology tends to consolidate primarily in this region. In contrast, apical bifurcations showed a lower incidence, approximately 25% (p = 0.044), which is consistent with previous reports (26 - 28). Although less common, apically located divisions present a higher degree of difficulty during endodontic canal preparation, whereas cervical bifurcations pose an increased risk of furcal perforation (12 , 13). These findings reinforce the value of CBCT, as it enables detailed visualization of root morphology and helps identify anatomical variations. Recognizing these variations can enhance treatment planning and improve prognosis in clinical practice. However, despite its advantages, CBCT has some limitations, albeit minor, such as image noise caused by metallic materials or patient movement, which can affect interpretation quality. Nevertheless, it provides comprehensive and accurate information, significantly enhancing diagnostic precision (29 , 30). Finally, we highlight that this study, with a relevant sample size and a reproducible methodology, is one of the first reports on biradicular canines in Bolivia, providing valuable information to the dental community and expanding knowledge of root anatomy in the Latin American population, although further studies are still needed.

## Conclusions

The prevalence of mandibular biradicular canines evaluated using CBCT was 1.7%, which is within the range reported in international studies, confirming that this is an uncommon yet clinically relevant anatomical variant. A higher prevalence was observed in women, and a consistent finding was the buccolingual orientation of the roots, an important detail, as it may go unnoticed on two-dimensional radiographs due to structural superimposition. Most bifurcations were in the middle third of the root, and no significant association was found regarding unilateral or bilateral distribution, the latter being less frequent. These results underscore the importance of recognizing this anatomical variation during dental treatment planning, particularly in endodontic, orthodontic, and surgical procedures, to prevent complications and improve treatment prognosis.

## Figures and Tables

**Table 1:** Sample initial characteristics.

Sex	n	AgeMean	SD	p
Female	625	44.40	17.90	0.680
Male	500	43.96	17.85	

T-test

**Table 2:** Prevalence of biradicular canine regarding sex and side.

Sex		Biradicular canine		Total	p
		Absent	Present		
Female	n	582	14	596	0.045*
	%	97.7	2.3	100	
Male	n	471	4	475	
	%	99.2	0.8	100	
Total	n	1053	18	1071	
	%	98.3	1.7	100	
Side		Biradicular canine		Total	p
		Absent	Present		
Right	n	1053	18	1071	0.305
	%	98.3	1.7	100	
Left	n	1048	14	1062	
	%	98.7	1.3	100	
Total	n	2101	32	2133	
	%	98.5	1.5	100	

*SignificantFisher exact test

**Table 3:** Association between sex, the affected side, level of furcation, and position of the biradicular canine.

Sex		Side		Total	p
		Unilateral	Bilateral		
Female	n	12	2	14	0.554
	%	85.7	14.3	100	
Male	n	3	1	4	
	%	75	25	100	
Total	n	15	3	18	
	%	83.3	16.7	100	
Sex		Level of furcation			
		Median	Apical		
Female	n	12	2	14	0.044
	%	85.7	14.3	100	
Male	n	3	1	4	
	%	75	25	100	
Total	n	15	3	18	
	%	83.3	16.7	100	
Sex		Position			
		Bucco-Lingual	Mesio-Distal		
Female	n	14	0	14	
	%	100	0	100	
Male	n	4	0	4	
	%	100	0	100	
Total	n	18	0	18	
	%	100	0	100	

*SignificantFisher exact test

## Data Availability

The data that support the findings of this study are available from the corresponding author upon reasonable request.

## References

[1] Aljanakh MD (2023). Restorative and Endodontic Management of a Mandibular Canine With Two Roots and Two Canals: A Case Report. Cureus.

[2] Martins JNR; Worldwide Anatomy Research Group; Versiani MA (2024). Worldwide Anatomic Characteristics of the Mandibular Canine-A Multicenter Cross-Sectional Study with Meta-Analysis. J Endod.

[3] Wolf TG, Anderegg AL, Yilmaz B, Campus G (2021). Root Canal Morphology and Configuration of the Mandibular Canine: A Systematic Review. Int J Environ Res Public Health.

[4] Li J, Parada C, Chai Y (2017). Cellular and molecular mechanisms of tooth root development. Development.

[5] Luan X, Ito Y, Diekwisch TG (2006). Evolution and development of Hertwig’s epithelial root sheath. Dev Dyn.

[6] Aminsobhani M, Sadegh M, Meraji N, Razmi H, Kharazifard MJ (2013). Evaluation of the root and canal morphology of mandibular permanent anterior teeth in an Iranian population by cone-beam computed tomography. J Dent (Tehran).

[7] Piskórz M, Futyma-Gąbka K, Różyło-Kalinowska I (2023). Prevalence of two-rooted and one-rooted mandibular canines with two root canals in Poland, assessed using CBCT: A preliminary study. Dent Med Probl.

[8] Alhumaidi AM, Mirza MB, Karobari MI, Abuelqomsan MA, Hashem Q, Aldaijy MT, Albarr NY, Aldaijy RT, Al Moaleem M (2025). Classifying the internal anatomy of anterior teeth in the Yemeni population using two systems: a retrospective CBCT study. Odontology.

[9] Almohaimede AA, Alqahtani AA, Alhatlani NM, Alsaloom NS, Alqahtani SA (2021). Interpretation of Root Canal Anatomy of Maxillary and Mandibular Permanent Canines in Saudi Subpopulation: A Cone-Beam Computed Tomography (CBCT) Study. Int J Dent.

[10] Karobari MI, Parveen A, Mirza MB, Makandar SD, Nik Abdul Ghani NR, Noorani TY, Marya A (2021). Root and Root Canal Morphology Classification Systems. Int J Dent.

[11] Iqbal A, Karobari MI, Alam MK, Khattak O, Alshammari SM, Adil AH (2022). Evaluation of root canal morphology in permanent maxillary and mandibular anterior teeth in Saudi subpopulation using two classification systems: a CBCT study. BMC Oral Health.

[12] Plascencia H, Gascón G, Ramírez B, Díaz M (2017). Mandibular Canines with Two Roots and Two Root Canals: Case Report and Literature Review. Case Rep Dent.

[13] Okumus M, Coban G (2022). Root canal morphology of mandibular canines in a Turkish subpopulation: a CBCT study. Odovtos Int J Dent Sci.

[14] Lambrianidis T, Lyroudia K, Pandelidou O, Nicolaou A (2001). Evaluation of periapical radiographs in the recognition of C-shaped mandibular second molars. Int Endod J.

[15] Soleymani A, Namaryan N, Moudi E, Gholinia A (2017). Root Canal Morphology of Mandibular Canine in an Iranian Population: A CBCT Assessment. Iran Endod J.

[16] Shaiban AS, Zayed AM, Abdullah AA, Alshaya KH, Alobaid MA, Al Malwi AA (2023). Analysis of Root Canal Characteristics in Permanent Canine Teeth Among 270 Saudi Subjects: A Cone-Beam Computed Tomography Study. Med Sci Monit.

[17] Zhao Y, Dong YT, Wang XY, Wang ZH, Li G, Liu MQ, Fu KY (2014). [Cone-beam computed tomography analysis of root canal configuration of 4 674 mandibular anterior teeth]. Beijing Da Xue Xue Bao Yi Xue Ban.

[18] Pan JYY, Parolia A, Chuah SR, Bhatia S, Mutalik S, Pau A (2019). Root canal morphology of permanent teeth in a Malaysian subpopulation using cone-beam computed tomography. BMC Oral Health.

[19] Mirza MB (2022). Evaluation of root and canal morphologies of permanent canines in a Saudi Arabian sub population using cone-beam computed tomography. J Dent Sci.

[20] Martins JNR, Marques D, Francisco H, Caramês J (2018). Gender influence on the number of roots and root canal system configuration in human permanent teeth of a Portuguese subpopulation. Quintessence Int.

[21] Alenezi MA, Al-Nazhan SA, Al-Omari MA (2022). Three-dimensional evaluation of root canal morphology of maxillary first premolars: Micro-computed tomographic study. Saudi Dent J.

[22] Zhang Q, Ran X, Zhao Y, Qin K, Zhang Y, Cui J, Yang Y (2024). Bilateral Symmetrical Mandibular Canines with Two Roots and Two Separate Canals: A Case Report and Literature Review. Curr Med Imaging.

[23] Ahmed HMA, Versiani MA, De-Deus G, Dummer PMH (2018). A new system for classifying root and root canal morphology. Int Endod J. 2017 Aug;50(8):761-770. Erratum in: Int Endod J.

[24] Roy DK, Cohen S, Singh VP, Marla V, Ghimire S (2018). Endodontic management of mandibular canine with two roots and two canals: a rare case report. BMC Res Notes.

[25] Kulkarni NR, Kamat SB, Hugar SI, Nanjannawar GS (2016). Mandibular Canine with Two Roots and Two Root Canals - A Rare Case. J Clin Diagn Res.

[26] Versiani MA, Pécora JD, Sousa-Neto MD (2011). The anatomy of two-rooted mandibular canines determined using micro-computed tomography. Int Endod J.

[27] Beltes P, Kantilieraki E, Kalaitzoglou ME, Beltes C, Angelopoulos C (2019). Mandibular canines with additional roots: An ex vivo study of the external and internal morphology. Aust Endod J.

[28] Rahimi S, Milani AS, Shahi S, Sergiz Y, Nezafati S, Lotfi M (2013). Prevalence of two root canals in human mandibular anterior teeth in an Iranian population. Indian J Dent Res.

[29] Mustafa M, Karobari MI, Al-Maqtari AAA, Abdulwahed A, Almokhatieb AA, Almufleh LS (2025). Investigating root and canal morphology of anterior and premolar teeth using CBCT with a novel coding classification system in Saudi subpopulation. Sci Rep.

[30] Kolarkodi SH (2023). The importance of cone-beam computed tomography in endodontic therapy: A review. Saudi Dent J.

